# Oral Ingestion of Transgenic RIDL *Ae. aegypti* Larvae Has No Negative Effect on Two Predator *Toxorhynchites* Species

**DOI:** 10.1371/journal.pone.0058805

**Published:** 2013-03-20

**Authors:** Oreenaiza Nordin, Wesley Donald, Wong Hong Ming, Teoh Guat Ney, Khairul Asuad Mohamed, Nor Azlina Abdul Halim, Peter Winskill, Azahari Abdul Hadi, Zulkamal Safi'in Muhammad, Renaud Lacroix, Sarah Scaife, Andrew Robert McKemey, Camilla Beech, Murad Shahnaz, Luke Alphey, Derric David Nimmo, Wasi Ahmed Nazni, Han Lim Lee

**Affiliations:** 1 Medical Entomology Unit, Institute for Medical Research, Jalan Pahang, Kuala Lumpur, Malaysia; 2 Department of Zoology, University of Oxford, Oxford, United Kingdom; 3 Oxitec Limited, Abingdon, Oxford, United Kingdom; 4 Medical Research Council Centre for Outbreak Analysis and Modelling, Department of Infectious Disease Epidemiology, Imperial College, London, United Kingdom; University of Texas at El Paso, United States of America

## Abstract

Dengue is the most important mosquito-borne viral disease. No specific treatment or vaccine is currently available; traditional vector control methods can rarely achieve adequate control. Recently, the RIDL (Release of Insect carrying Dominant Lethality) approach has been developed, based on the sterile insect technique, in which genetically engineered ‘sterile’ homozygous RIDL male insects are released to mate wild females; the offspring inherit a copy of the RIDL construct and die. A RIDL strain of the dengue mosquito, *Aedes aegypti*, OX513A, expresses a fluorescent marker gene for identification (DsRed2) and a protein (tTAV) that causes the offspring to die. We examined whether these proteins could adversely affect predators that may feed on the insect. *Aedes aegypti* is a peri-domestic mosquito that typically breeds in small, rain-water-filled containers and has no specific predators. *Toxorhynchites* larvae feed on small aquatic organisms and are easily reared in the laboratory where they can be fed exclusively on mosquito larvae. To evaluate the effect of a predator feeding on a diet of RIDL insects, OX513A *Ae. aegypti* larvae were fed to two different species of *Toxorhynchites* (*Tx. splendens* and *Tx. amboinensis*) and effects on life table parameters of all life stages were compared to being fed on wild type larvae. No significant negative effect was observed on any life table parameter studied; this outcome and the benign nature of the expressed proteins (tTAV and DsRed2) indicate that *Ae. aegypti* OX513A RIDL strain is unlikely to have any adverse effects on predators in the environment.

## Introduction

Epidemic dengue fever and dengue haemorrhagic fever (DHF) have emerged as major global public health problems in recent decades. According to the World Health Organization (WHO) dengue epidemiology is rapidly worsening [1] with increased frequency of outbreaks and expansion into new geographical areas. This expansion has partly been driven by the rapid increase of the global range of *Aedes aegypti* in the last few decades. *Ae. aegypti* was eliminated from many areas of the world 40–50 years ago through the use of DDT but is now distributed more widely than it was before control began, and is now present in large urban areas where a greater number of people than in the past are at risk [2]. Failure to control the spread of *Ae. aegypti* has led to the re-emergence of the disease in many areas across the globe.

As for malaria there is no licensed vaccine for dengue, though several candidates are in various stages of trials. Unlike malaria, for dengue there are no specific therapeutic or prophylactic drugs. Control has therefore focused on the mosquito; however bed nets, widely used against malaria, are relatively ineffective for dengue as *Ae. aegypti* bites primarily in the day time [3]. Current control methods are therefore based primarily on breeding site elimination with larvicides or other methods, and some use of adulticides. These methods have not proven adequate to prevent epidemic dengue in any but the most favourable of circumstances [4], [5], [6]. More and better options for controlling *Ae. aegypti* are urgently required. The sterile insect technique (SIT) has been used for decades to control several insect pest species [7]. The technique mainly uses irradiation to sterilise the insects, however this appears to cause significant fitness effects on mosquitoes that prevent its widespread use for vector control [8], [9]. The release of insects with dominant lethality (RIDL) is a new method to control insects that replaces irradiation with the insertion of a conditional lethal gene [10], [11], [12]. The expression of the RIDL system is dependent on the absence of a suppressor (tetracycline) in the insects' diet. In the presence of tetracycline, expression is suppressed and the insects survive. The mechanism of sterility is the transmission to the progeny of a lethal transgene; equivalent to the transmission of radiation-induced dominant lethal mutations in classical SIT.

A line of *Ae. aegypti* (OX513A) has been developed that causes death of the mosquitoes at L4/pupae stage in the absence of tetracycline [13]. The protein tTAV is a codon optimised version of tTA for more efficient expression in insects [14] and is part of the positive feedback system in RIDL, developed from the well-known tet-off gene expression system [15], [16]. This system has been widely used in gene expression studies in mice [17], [18], [19], rats [20] and many different mammalian cell lines [21]. Only high level intra-cellular expression of tTA causes cell death, presumably *via* transcriptional squelching [15], [20], [22] and the levels that may be ingested by a predator eating mosquitoes would be predicted to have no potential adverse effects. The *Ae. aegypti* line also expresses a fluorescent marker protein DsRed2, for identification. DsRed2 is a member of the GFP superfamily of fluorescent proteins [23], [24]. DsRed2 has been used in a wide variety of transgenic organisms, including plants, insects and mice and is not expected to be harmful by ingestion [25], [26], [27], [28], [29].

As both tTA and DsRed2 are introduced proteins expressed in OX513A *Ae. aegypti* larvae, we asked the question if they could adversely affect potential predators that ingested the insect. Choice of a representative from the guild of potential predators in the invertebrate ecosystem is important as not all predator species can be tested in the laboratory [30], [31], consequently surrogate test species have to be used that are representative of potential non-target organisms in the field. An ideal surrogate test species would be amenable to testing under laboratory conditions, available, ecologically relevant, and sensitive to the substance under test, and in the case of oral exposure studies be capable of consuming significant quantities of test substance without gastric imbalance. *Toxorhynchites* is a predatory mosquito whose larvae feed on other aquatic invertebrates including mosquito larvae and has been used in attempts to control mosquitoes [32], [33], [34], [35]. *Ae. aegypti* tends to breed in small pools of water in and around human habitation as the females almost exclusively feed on humans [36]. These breeding sites are predominantly man made, plastic containers, water storage containers, discarded rubbish etc. fed from rain water, or human-filled [37], [38], [39]. These types of breeding sites do not contain many predators and to our knowledge there is no predator that exclusively feeds on *Ae. aegypti* larvae [40], [41]. However *Toxorhynchites* can be fed exclusively on *Ae. aegypti* larvae, is easily maintained in the laboratory, is a natural predator of *Aedes* species and therefore represented a credible test species from the guild of predators.

To test if *Toxorhynchites* was affected by feeding on OX513A larvae two different species, *Tx. splendens* and *Tx. amboinensis*, were fed on each of several types of *Ae. aegypti* larvae: wild type (WT), OX513A reared off tetracycline and OX513A reared on tetracycline. OX513A when reared off tetracycline expresses the tTAV protein at a higher level than when reared in the presence of tetracycline (on-tet); these two treatments therefore provide different doses of tTAV. *Toxorhynchites* life table parameters of larval development, survival, fecundity and size were compared between the different treatments.

## Results

**Figure 1:**
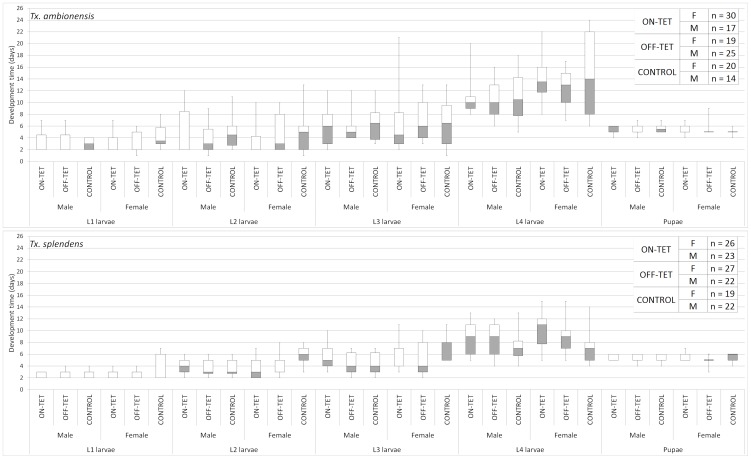
Box plot summary of development time (days) of different life stages. Minimum and maximum development time are shown by vertical lines, the upper and lower quartiles are shown by the bottom and top of box respectively, the median is represented by horizontal line inside box; where the median value is the same as the upper and lower quartile the top of the gray or the bottom of the white box represents the median. Individuals for which sex could not be determined due to death prior to adult emergence were excluded from this analysis, these unclassified individuals represented at most 43% of each type and averaged 26.6% (see Table S1 for complete dataset. There was a significant difference in L4 larval development time between *Tx. amboinensis* and *Tx. splendens*.

There was no significant difference between larval or pupal development time for any of the treatments or the two different species of *Toxorhynchites* (Figure 1 and Table S1). There was a significantly longer development time of L4 male and female larvae (identified from individuals that survived to adults) in the control fed group of *Tx. amboinensis* compared to *Tx. splendens*. However there was a trend for longer development of L4 larvae in *Tx. amboinensis* throughout the treatments suggesting that this species has a slightly longer development time for this stage. Larvae that did not survive to adulthood could not readily be identified as male or female. The survival of each life stage in the unclassified group was more variable due to some larvae remaining at a particular developmental stage for longer than normal before death. The reason for this delayed development is unknown but the proportion surviving to adults was not significantly different between treatment groups for *Tx. splendens* (χ^2^ = 4.0, d.f.  = 2, p = 0.13). However for *Tx. amboinensis* the control treatment did have significantly less overall survival to adults than the OX513A on-tet or OX513A off-tet treatments (χ^2^ = 6.4, d.f. = 1, p<0.05, data not shown). This was due to one of the repeats of the control treatment having significantly lower survival than the other two repeats. The cause of this low survival is unknown and was not reflected in other treatments set up at the same time. Excluding the results of this repeat removed the significant difference in survival so we conclude that this result was due to one aberrant control treatment.

In both *Toxorhynchites* species there were significantly more larvae consumed in the off tetracycline treatments; *Tx. amboinensis* (t = 9.2, p<0.001) and Tx. s*plendens* (t = 8.3, p<0.001). However OX513A larvae when reared off tetracycline die at L4/pupal stage, to compensate more third instar (L3) larvae were used to provide the equivalent mass of fourth instar (L4) larvae that would have been used. This is reflected in the number of larvae consumed by L4 *Toxorhynchites*, on average L3 *Ae. aegypti* larvae are about one-third the weight of L4 larvae (in a parallel experiment, L3 larvae averaged 0.830 µg (+/−0.017ug) wet weight and L4 larvae 2.995 µg (+/−0.024)) and the number of L4 larvae consumed in the off-tet experiment was approximately 3–4 times on-tet and control experiments (data not shown). Therefore we attribute the variance in number of larvae consumed to the different feeding regimes used between the treatments.

*Tx. amboinensis* females reared on WT larvae consumed significantly more larvae than females fed on OX513A larvae reared on-tetracycline (t = −3.3, p<0.002). We don't know why this treatment consumed more larvae but there was no significant difference in any other parameters.

**Figure 2:**
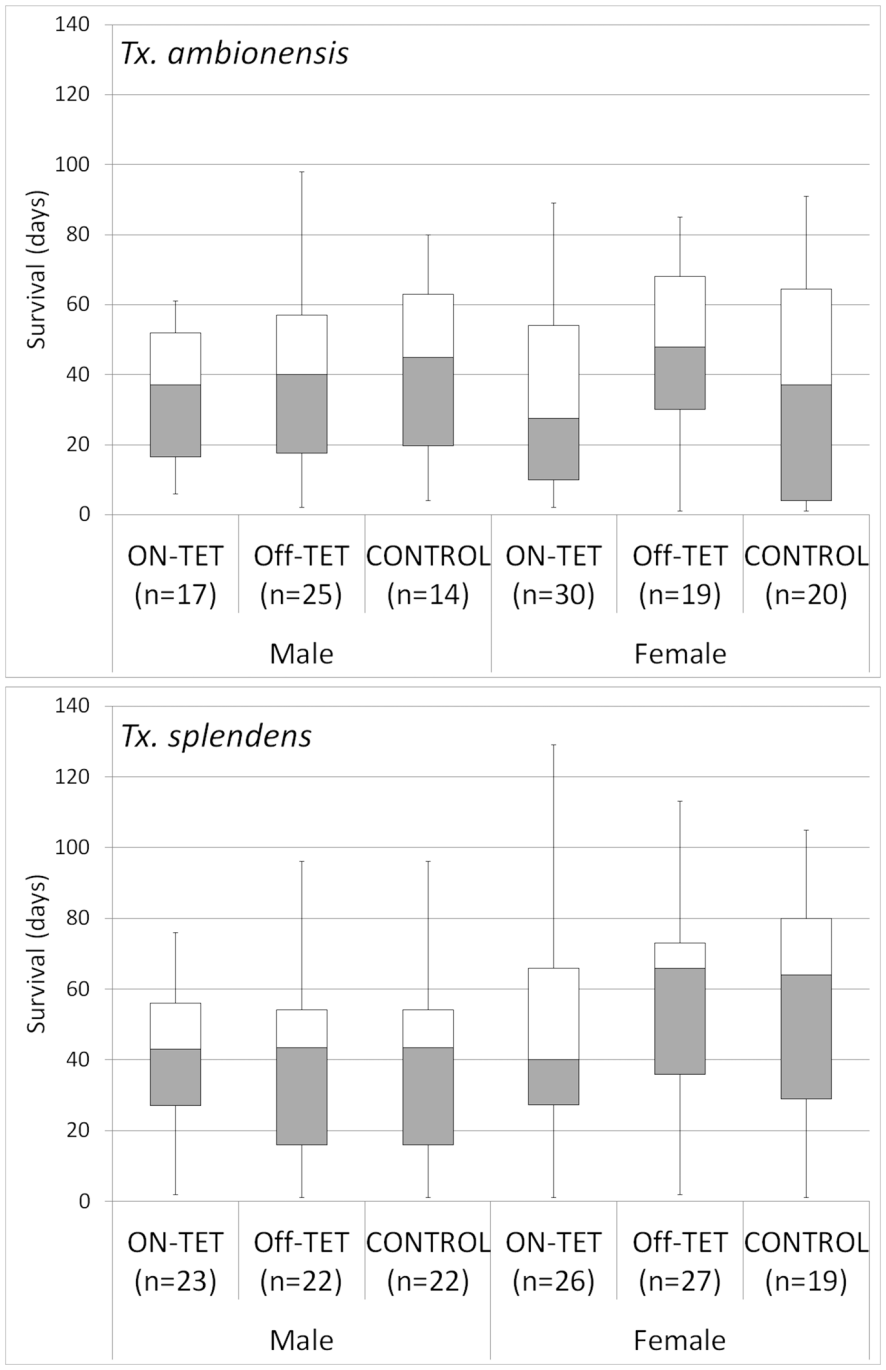
Box plot of adult survival (days). Minimum and maximum survival are shown by vertical lines, the upper and lower quartiles are shown by the bottom and top of box respectively, the median is represented by horizontal line inside the box. No significant difference was observed for adult survival between treatments or species.

Adult survival is summarised in Figure 2. There was no significant difference in the survival of male (*Tx. splendens* χ^2^ = 1.0, d.f. = 2, p = 0.60 and *Tx. amboinensis* χ^2^ = 0.5, d.f. = 2, p = 0.76 ) and female (*Tx. splendens* χ^2^ = 2.6, d.f. = 2, p = 0.28 and *Tx. amboinensis* χ^2^ = 2.5 d.f. = 2, p = 0.29) adults across treatment groups for both species of *Toxorhynchites*.

The number of eggs laid per female for both *Toxorhynchites* species across all treatment groups did not significantly differ (see Table S1). However because of the large variation in egg production between individual females only relatively strong effects would likely have been detected by this assay.

**Figure 3:**
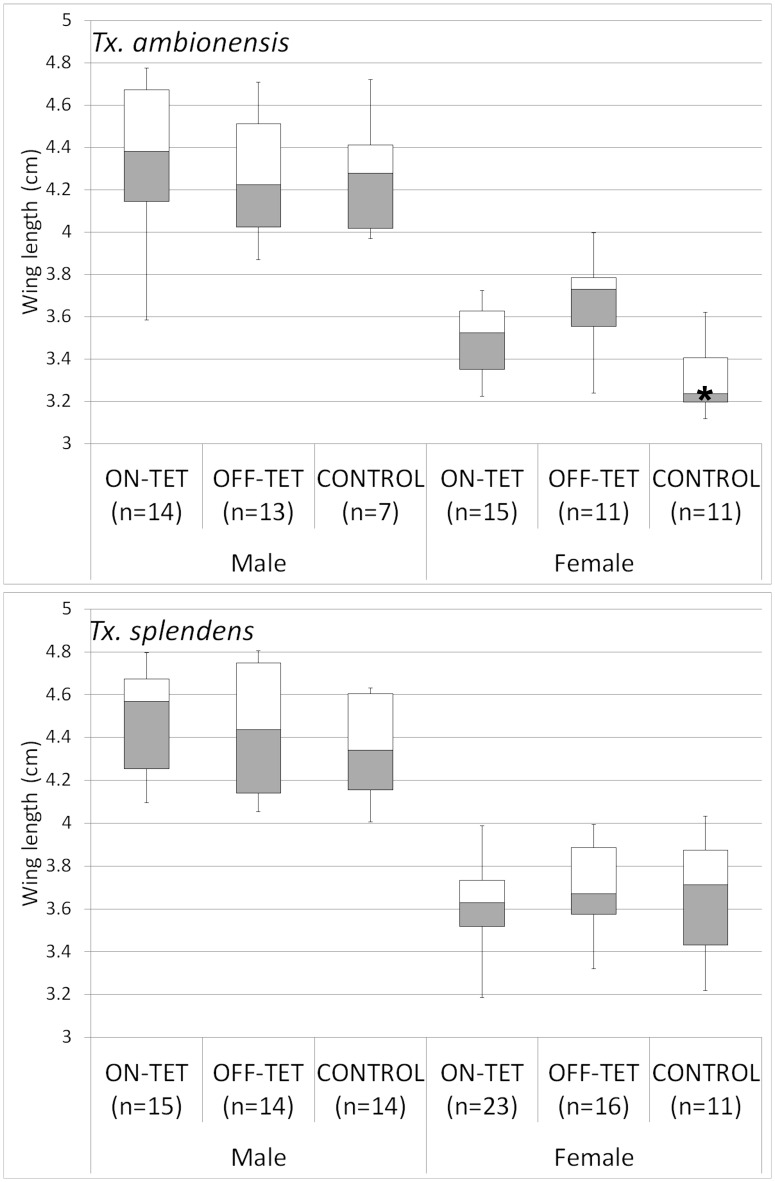
Wing length results summarised in a box plot. Wing lengths for each adult are the average of the left and right wing measurements. Minimum and maximum wing lengths are shown by vertical lines, the upper and lower quartiles are shown by the bottom and top of box respectively, the median is represented by the horizontal line inside the box. Females that were fed on WT larvae (Control) were significantly smaller than females that were fed on RIDL larvae reared off-tetracycline (OFF-TET), highlighted by asterisk. No other significant differences were observed.

The size of the *Toxorhynchites* adults was determined from wing length measurements (Figure 3) and the only significantly different result was females from *Tx. amboinensis* control treatment were smaller than females from the off-tet treatment group (t = −3.1, p = 0.012). We are unsure why this group was significantly smaller but the difference is small and this is the same control group where one repeat had low survival. The females from this low survival group were smaller than usual however removing them from the analysis does not change the overall result; t = 2.06, p = 0.048.

We also examined *Toxorhynchites* fed on RIDL larvae for presence of the RIDL transgene by testing the adults by PCR. This test looked for unexpected persistence of the transgene, which might have indicated horizontal gene transfer (HGT), among other possibilities. A total of 121 adults gave DNA of sufficient quality to test, as judged by amplification of a control DNA fragment; none were positive for the transgene. On average each *Toxorhynchites* larvae consumed 431 RIDL larvae, thus there were over 52,000 events that had the potential for horizontal gene transfer; however none were detected in the adults tested. Comparative genomics and other considerations imply that HGT rates are expected to be extremely low, many orders of magnitude below the limits of sensitivity of this experiment [42], [43]. That we detected no such events is therefore not surprising but it does suggest that no unrecognised high-efficiency mechanism for DNA persistence or transfer exists in this case.

## Discussion

In a control programme, RIDL male mosquitoes are released into the environment and subsequently mate with wild females. Those wild females that have mated a RIDL male may then lay eggs and the resulting larvae die before adulthood due to the lack of tetracycline in the environment. Predators that feed on these larvae or pupae are then exposed to the transgene and its products, e.g. encoded protein(s), raising the question of whether this exposure might have any potential adverse effects on such predators.

The positive feedback RIDL system is repressed by tetracycline, however off tetracycline there is increased expression of mRNA, up to 672 fold increase in homozygotes [14] and in OX513A death occurs in L4 larvae and pupae [13]. The aim of feeding *Toxorhynchites* on OX513A larvae was to investigate any effects of the transgene and/or the marker (DsRed2) on development. A significant advantage of using this predator was the ability to feed it exclusively on mosquito larvae (100% of diet) without expecting this restricted diet itself to have a negative effect on development or other parameters measured.

In separate experiments, OX513A larvae reared on-tet or off-tet were used, with equivalent wild type controls. This allows us to identify potential effects of high level expression of tTAV – produced in OX513A under off-tet conditions only – from other potential effects of the transgene, the only other obvious difference between the two treatments being the presence of tetracycline. In fact no negative effects were detected from feeding larvae reared either on or off-tet.

The transcriptional activator tTA has been used in several mammalian species and does not have any adverse effects unless expressed in large amounts and in various tissues [20]. Numerous experimental uses of tTA show that the effect of expression is cell-autonomous, i.e. only affects those cells in which the tTA protein is expressed. Dietary tTAV is not expected to have an effect due to considerations of the amount of biologically active protein potentially available and the lack of a mechanism for intact uptake of this protein to a relevant subcellular compartment. DsRed2 belongs to family of fluorescent proteins which are part of a group of proteins from the *Anthozoa* species. The protein family has been widely used in a variety of species, including plants, insects and mammals without adverse effects as well as subject to an evaluation by the FDA for food safety [29]. These factors lead to a lack of potential hazard from the ingestion by predators eating mosquito larvae or adults.

Furthermore, mosquitoes in aggregate are not a major diet component for vertebrates [40], [41], and *Ae. aegypti* is a relatively low-density species even in areas where it is epidemiologically important because of its anthropophagic nature. Each of these factors further indicates very low maximum exposure for predators and scavengers in the field relative to the 100% diet used in the experiments reported here.

## Conclusion

Both *Tx. splendens* and *Tx. amboinensis* showed no adverse effects of being fed OX513A larvae either reared on tetracycline or off tetracycline compared to being fed non-transformed *Ae. aegypti* larvae. Although some significant variation was observed, partly due to species, no evidence was found that indicated the OX513A larvae had adverse effects on the development, fecundity and longevity of two species of *Toxorhynchites* larvae. No transfer of transgene DNA between the species was observed. These results show that *Ae. aegypti* OX513A RIDL strain is unlikely to have any adverse effects on predators in the environment.

## Materials and Methods

Two different strains of *Toxorhynchites* were used, *Tx. splendens* originally isolated from Thailand and *Tx. amboinensis* originally isolated from Hawaii. Both of these species have been maintained at the Institute for Medical Research (IMR), Kuala Lumpar for 811 generations and 834 generations for *Tx. splendens* and *Tx. amboinensis* respectively. Both species were maintained at 25°C (+/−1°C) with 80% (+/−10%) humidity and fed on *Ae. aegypti* WT larvae. The *Aedes aegypti* transgenic strain used in this experiment was OX513A [13], produced in 2002 and subsequently made homozygous for the transgene. OX513A had been reared in the lab for more than 60 generations and maintained at 27^o^C (+/−1°C) and 80% (+/−10%) relative humidity. The wild type *Ae. aegypti* strain was isolated from Jinjang, Selangor, Malaysia in 1960. The OX513A insertion was originally in a Rockefeller strain background [13]. Prior to this study, that OX513A had been backcrossed into this Malaysian wild type strain background for 5 generation and then made homozygous for OX513A; ∼97% of its genome is expected to derive from the Malaysian wild type strain [44], [45].

Three treatments were used; *Toxorhynchites* fed on WT, OX513A reared on tetracycline (BioBasic Inc, 64-75-5) and OX513A reared without tetracycline. Tetracycline was added at a concentration of 30 µg/ml after hatching and not refreshed. Each treatment had twenty repeats each containing a single *Toxorhynchites* larva in a small circular plastic cup (7.5cm deep, 8.5cm diameter) half filled with tap water. Each treatment set was prepared simultaneously for both species of *Toxorhynchites* and three repeats were independently performed.

*Ae. aegypti* larval preparation for feeding *Toxorhynchites*; the eggs of *Ae. aegypti* for all treatments were hatched under vacuum and all larvae were reared at 1 larvae per ml and with equal amounts of food (Tetramin® flake fish food). The *Ae. aegypti* larvae were maintained at a level of 20 per *Toxorhynchites* larva by replenishing those that had been eaten daily. The larvae that were replenished were matched in developmental stage to the *Toxorhynchites* larvae. In the case of OX513A larvae that were reared off-tetracycline there were few L4 larvae available, many die at this stage and *Toxorhynchites* does not feed on dead larvae, so an equivalent mass of L3 larvae was added.

The duration of each developmental stage was recorded daily. The *Toxorhynchites* larvae from each treatment that survived to pupae were placed into separate cages (23cm X 23cm X 23cm). Females were provided with a plastic container filled with tap water for egg laying and 10% sucrose solution (including 1% vitamin B complex). Females were provided with 5–8 males from the stock colony. The number of eggs was recorded daily along with survival. After death the wing length was recorded [46].

PCR for presence of transgene in adult *Toxorhynchites*: Genomic DNA was extracted from single adult or late larval individuals using the GeneJET genomic DNA purification kit from Fermentas, according to the kit protocol. Genomic DNA was diluted 1 in 20 with water, and 1 µl of this dilution used in a 20 µl PCR reaction using Dreamtaq polymerase and buffer (Fermentas). Primers 38DrosF (ATGAGCAATTAGCATGAACGTT) and 48HspdiagR (GCAGATTGTTTAGCTTGTTCAGC) were used to amplify a fragment of the OX513 transgene (1233bp product). An OX513A RIDL *Ae. aegypti* control gDNA was included in each PCR reaction along with water negative control. In addition, all samples were amplified with primers 894AeMuAcF (CAGGGTGTGATGGTCGGTATGGG) and 895AeMuAcR (CCCAGGAAGGATGGCTGGAAGAG), which amplify endogenous muscle actin (660bp product), to check gDNA quality. For both primer sets, PCR conditions were: 94°C for 2 min's followed by 10 cycles of 94°C for 15s, 55°C (decreasing by 0.5°C per cycle) for 40s and 72°C for 1 min; followed by 25 cycles of 94°C for 15s, 50°C for 40s and 72°C for 1 min, with a final elongation step of 72°C for 7 min's.

Results were statistically analysed using STATA (version 12, College Station, TX, USA). All variables were assessed for normality. Experimental repeats were examined to determine if they could be combined for the final analysis. Differences in wing length across treatment group were compared using ANOVA and t-test. The non-parametric equivalents, Kruskal-Wallis and Mann-Whitney tests, were used to compare egg-counts and longevity across treatment groups. The proportion of individuals surviving to become adults was examined using Chi-squared test.

## Supporting Information

Table S1 **Summary of results.** The table shows the mean and (in brackets) standard deviation for each of the parameters measured, for *Tx. spendens* and *Tx. amboinensis* fed on WT (control), OX513A reared off tetracycline (OX513A OFF TET) and OX513A reared on tetracycline (OX513A ON TET). The results for females (F), males (M) and those individuals that did not survive to adults for identification of sex (U) are shown for each treatment. Because of the large variation in results from larvae and pupae that died (U) they have been excluded from statistical analysis; except for overall larval survival. Significantly different results discussed in the text are indicated by symbols; * and # for significantly different results within species and ¥ for between species.
(DOC)
