# Supplementary material for: Oral Ingestion of Transgenic RIDL Ae. aegypti Larvae Has No Negative Effect on Two Predator Toxorhynchites Species
Source: PLoS One. 2013 Mar 20;8(3):e58805. doi: 10.1371/journal.pone.0058805 (PMC3604150; doi:10.1371/journal.pone.0058805)
Supplement: Table S1 — Summary of results. The table shows the mean and (in brackets) standard deviation for each of the parameters measured, for Tx. spendens and Tx. amboinensis fed on WT (control), OX513A reared off tetracycline (OX513A OFF TET) and OX513A reared on tetracycline (OX513A ON TET). The results for females (F), males (M) and those individuals that did not survive to adults for identification of sex (U) are shown for each treatment. Because of the large variation in results from larvae and pupae that died (U) they have been excluded from statistical analysis; except for overall larval survival. Significantly different results discussed in the text are indicated by symbols; * and # for significantly different results within species and ¥ for between species. (DOC) [file pone.0058805.s001.doc]

|  |  |  | Average development time (days) | | | | | Average survival (days) | Average of other parameters | | |
| --- | --- | --- | --- | --- | --- | --- | --- | --- | --- | --- | --- |
|  |  | n | L1 | L2 | L3 | L4 | Pupae | Adults | Eggs | Wing length (cm) | Larval consumption |
| *Tx. amboinensis* | | | | | | | | | | | |
| Control | F | 19 | 4.10 (1.80) | 4.85 (3.07) | 6.40 (3.36) | 14.25 (6.73)¥ | 5.05 (0.60) | 40.55 (42.67) | 131 (75) | 3.32 (0.17) [n=11]* | 389 (56)# |
| Control | M | 22 | 3.07 (0.92) | 4.64 (2.50) | 6.43 (2.74) | 10.86 (4.00)¥ | 5.64 (0.74) | 42.36 (23.89) | - | 4.24 (0.24) [n=7] | 347 (59) |
| Control | U | 19 | 2.69 (0.93) | 2.15 (1.12) | 3.46 (1.27) | 15.04 (5.18) | 3.22 (2.44) | - | - | - | 340 (113) |
| OX513A OFF TET | F | 27 | 3.26 (1.76) | 4.37 (2.91) | 6.79 (3.08) | 12.16 (2.81) | 5.47 (1.07) | 46.74 (24.31) | 138 (90) | 3.67 (0.21) [n=11]* | 649 (102)* |
| OX513A OFF TET | M | 22 | 3.08 (1.55) | 3.92 (2.31) | 5.84 (2.12) | 10.60 (2.89) | 5.32 (0.75) | 38.88 (27.31) | - | 4.22 (0.29) [n=13] | 544 (150)* |
| OX513A OFF TET | U | 11 | 2.63 (1.31) | 3.36 (1.22) | 5.23 (2.74) | 13.08 (6.50) | 2.50 (1.87) | - | - | - | 359 (250)* |
| OX513A ON TET | F | 26 | 2.83 (1.49) | 3.27 (2.41) | 6.03 (4.40) | 13.77 (3.50) | 5.37 (0.81) | 34.43 (26.23) | 93 (62) | 3.48 (0.18) [n=15] | 337 (54)# |
| OX513A ON TET | M | 23 | 3.18 (1.67) | 4.82 (3.61) | 6.12 (3.61) | 11.12 (3.50) | 5.53 (0.72) | 35.71 (18.13) | - | 4.25 (0.41) [n=14] | 321 (52) |
| OX513A ON TET | U | 11 | 3.23 (1.54) | 4.08 (2.97) | 9.58 (8.68) | 19.44 (16.69) | 5.00 (1.00) | - | - | - | 188 (88) |
| *Tx. splendens* | | | | | | | | | | | |
| Control | F | 20 | 3.42 (1.95) | 5.58 (1.50) | 7.47 (2.12) | 7.38 (2.34)¥ | 5.26 (0.65) | 55.32 (28.78) | 123 (66) | 3.59 (0.26) [n=11] | 318 (62) |
| Control | M | 14 | 3.36 (1.79) | 5.90 (1.85) | 6.41 (2.32) | 7.50 (2.50)¥ | 5.36 (0.58) | 36.32 (23.00) | - | 4.37 (0.22) [n=14] | 295 (39) |
| Control | U | 26 | 2.74 (1.37) | 5.37 (3.29) | 4.63 (3.98) | 5.61 (5.53) | 1.21 (1.96) | - | - | - | 234 (167) |
| OX513A OFF TET | F | 19 | 2.56 (0.85) | 3.85 (1.63) | 5.11 (2.74) | 9.00 (2.54) | 5.26 (0.76) | 57.11 (31.01) | 131 (62) | 3.71 (0.18) [n=16) | 503 (157)* |
| OX513A OFF TET | M | 25 | 2.45 (0.74) | 3.64 (1.47) | 4.36 (1.76) | 8.59 (2.54) | 5.36 (0.58) | 41.36 (28.62) | - | 4.44 (0.29) [n=14] | 493 (151)* |
| OX513A OFF TET | U | 16 | 2.64 (0.81) | 2.90 (1.91) | 2.73 (2.65) | 6.64 (5.90) | 2.45 (3.30) | - | - | - | 371 (295)* |
| OX513A ON TET | F | 30 | 2.38 (0.57) | 3.65 (1.62) | 5.50 (2.23) | 10.19 (2.62) | 5.54 (0.65) | 45.35 (29.56) | 126 (81) | 3.65 (0.24) [n=23] | 325 (35) |
| OX513A ON TET | M | 17 | 2.43 (0.51) | 4.13 (1.18) | 5.78 (2.26) | 8.83 (2.81) | 5.39 (0.50) | 40.61 (21.37) | - | 4.50 (0.24) [n=15] | 309 (34) |
| OX513A ON TET | U | 13 | 5.55 (8.54) | 2.09 (2.59) | 2.64 (3.93) | 4.80 (10.39) | 1.09 (2.43) | - | - | - | 168 (158) |
